# Primary healthcare approach to substance abuse management

**DOI:** 10.4102/safp.v63i1.5307

**Published:** 2021-05-26

**Authors:** Ramprakash Kaswa

**Affiliations:** 1 Department of Family Medicine and Rural Health, Walter Sisulu University, Mthatha, South Africa

**Keywords:** substance abuse, primary healthcare setting, screening tools, brief behavioural change counselling, co-morbidity

## Abstract

Substance abuse is common amongst patients attending primary healthcare settings. Despite the substantial impact on one’s health, substance abuse is often underdiagnosed by primary care providers owing to a lack of training and time for screening. Self-reported screening tools are easy to administer and efficient to make a substance abuse diagnosis in primary care settings. Comorbid mental illness and intimate partner violence are common amongst patients presenting with substance abuse in primary care. An early diagnosis and a brief behavioural change counselling are effective in managing substance abuse before it develops into dependency. A brief motivational communication rather than a confrontation during substance abuse screening, counselling and treatment is important to achieve optimum patient outcomes.

## Background

Substance abuse is a leading cause of preventable death that has become a global concern. About 5.0% of the global population between the ages of 15 years and 64 years has used at least one substance, and an estimated 0.6% of this population suffers from substance dependency.^1^ Harmful and hazardous use of tobacco, alcohol and other psychotic substances is defined as substance abuse.^2^ Globally, the most commonly abused substance is cannabis (dagga), followed by amphetamine and opioids.^1,3^

South Africa, like many parts of the globe, is experiencing an increased prevalence of substance abuse. The lifetime prevalence of substance abuse of the South African population is an estimated 13.3% for at least one substance.^1^ The prevalence of substance abuse amongst the South African population is more than twice the global average, and the rates of heavy alcohol drinking are the highest in the world.^4^ The most commonly abused substance in South Africa is alcohol, followed by tobacco and cannabis. Amongst young people, inhalant substances are more popular, and cannabis is the most commonly abused substance, followed by methamphetamine, amphetamine and heroin.^1,5^ An estimated 7% of the population use a narcotic substance during their lifetime.^1^

Recent literature has recognised the similarities between the course of illness of substance abuse and other common chronic conditions such as hypertension and diabetes mellitus. Like those illnesses, early identification of substance abusers when no dependency has developed yet can be managed successfully by less intensive treatment without any residual sequelae.^6^ Substance abuse is a complex health problem because of the chronic nature of the illness and is associated with multiple psychosocial problems. Substance abuse is prevalent across all social, racial, cultural, religious and gender spheres.^7^ Many research studies have reported the link between substance abuse and various physical and mental health problems, communicable diseases, intimate partner violence, child abuse, road traffic accidents and deaths from avoidable causes.^8,9^ Treatment, care and rehabilitation of substance abuse place a heavy burden on overstretched public health systems.^6^ The *Prevention of and Treatment for Substance Abuse Act No. 70, 2008* provides a comprehensive national response for the combating of substance abuse through prevention, early intervention, treatment and re-integration programme.

## Primary healthcare in the context of substance abuse

Primary healthcare (PHC) providers are well equipped to alleviate the substance abuse problem. Research has proved that even brief interventions from a PHC provider have a long-lasting positive impact on substance abusers’ behaviour.^10^ Primary healthcare settings are optimally positioned to reduce the burden of substance abuse by providing optimum patient-centred healthcare in their local context.^11^ This simple approach can reduce emergency department visits and hospitalisations and achieve improved outcomes of illness. Furthermore, the research demonstrated that adding effective technology-based interventions to PHC settings can improve adherence, self-management and even cessation of substance abuse.^5^

Prevention of substance abuse is more cost-effective than treatment, and PHC settings are well known for delivering effective preventive healthcare to the community. Family, social support systems and networks play a crucial role in substance abuse management. The psychosocial context can be part of the problem as well as the solution.^3^ The biopsychosocial approach of the substance abuse management of PHC providers can give effective support to the recovery of the patient.

**FIGURE 1:**
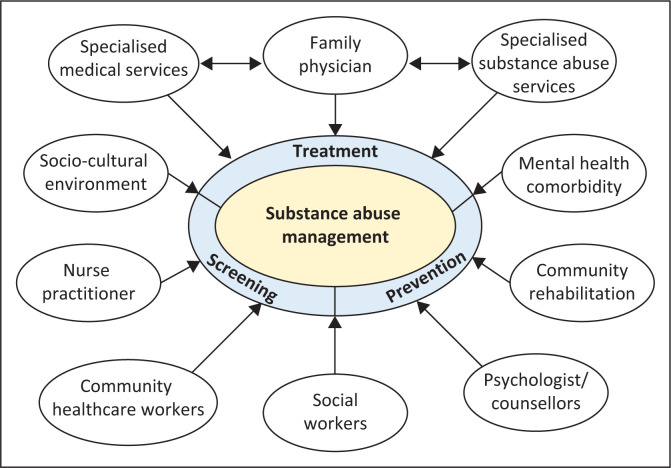
Theoretical framework of substance abuse management in primary healthcare settings.

Primary healthcare is an entry point for healthcare services, and most people who abuse substances are already visiting these facilities for different health-related issues. Managing substance abuse within the PHC context could improve access and the outcome of care.^12^ There is growing evidence of integrated management of substance abuse in PHC settings being more cost-effective than non-integrated care.^11,13^ Similar to chronic medical conditions, managing substance abuse requires long-term, continuous care, and PHC settings are well suited for these circumstances.^14^ Figure 1 shows the theoretical framework of substance abuse management in the PHC context.

## Aetiological factors

The aetiology of substance abuse is a complex interaction of individual personality, cognitive behaviour and sociocultural environment. Young people usually experiment firstly with alcohol and tobacco and subsequently move on to illicit substances. Thus, alcohol and tobacco have been defined as the gateway to substance abuse.^15^ The relevant aetiological factors are mainly defined under the following three categories:

- Biological
  ■ Male gender
  ■ Inherited genetic susceptibility to substance abuse
- Psychosocial
  ■ Personality traits, for example high impulsivity and aggressive behaviour
  ■ Comorbid mental health conditions, for example major depressive disorder, anxiety disorder, schizophrenia and post-traumatic stress disorder
  ■ Poor social skills
- Contextual
  ■ Parents and a family history of substance abuse
  ■ Peer substance abuse
  ■ Physical, emotional and sexual abuse
  ■ Dysfunctional family
  ■ Accessibility of substances in the community
  ■ Influence of print and online media

## Screening

Substance abuse screening is essential for comprehensive medical care and is an integral part of the patient’s medical history. Primary healthcare providers need to know about substance abuse, to make diagnoses, manage the condition and compile a follow-up plan for patients.^14^ Although a lack of training and time are well-known barriers to the screening of substance abuse in PHC settings,^3^ healthcare workers at the PHC facility have an opportunity of screening substance abuse on each encounter. Failure to identify the risk has potential consequences for the overall health outcomes.^16^

Screening for substance abuse can improve health outcomes, and universal screening may be justifiable in high-prevalence health settings.^17^ Self-report screening tools accurately identify substance abuse and are commonly implemented by PHC providers.^18^ Urine testing for substances should not be performed routinely but can be used to support a suspected diagnosis, assess for polysubstance use and monitor treatment response.^15^ Some of the commonly used self-reporting screening tools for substance abuse are the following:

- Alcohol Use Disorders Identification Test (AUDIT)
- The Alcohol, Smoking and Substance Involvement Screening Test (ASSIST)
- Brief Screener for Tobacco, Alcohol and Other Drugs (BSTAD)
- Cut down, Annoyed, Guilty, Eye-opener (CAGE)
- The Car, Relax, Alone, Forget, Family or Friends, Trouble (CRAFT) tool
- The Tobacco, Alcohol, Prescription medication and other Substance use (TAPS) tool:

## Prevention strategies

The best approach to substance abuse prevention that PHC providers can take is targeting the aetiological factors.^14^ Three levels of prevention have been described – primary, secondary and tertiary:

- Primary prevention involves interventions before the development of substance abuse. Different approaches to primary prevention of substance abuse are reported^19^:

**Individual-level Intervention:** (1) *Information dissemination*: Increase the knowledge of substances, their effect and consequences and promote anti-substance use attitude. (2) *Affective education*: Increase self-esteem and decision-making skills. (3) *Alternatives*: Provide variable alternatives to substance abuse. (4) *Resistance skills*: Develop skills for substance abuse resistance by increasing the awareness of the social influence of a substance. (5) *Personal and social skills training*: Increase personal development skills and develop assertive behaviour.

**Community-level Intervention**^20,21^: (1) Community engagement and mobilisation against substance abuse. (2) Reduce supply by restricted access of illicit substance. (3) Demand reduction by implementation of appropriate programmes into broader social welfare, health promotion and education programmes. (4) Implementation of national drug master plan.

- Secondary prevention of substance abuse includes screening and early intervention. Early identification of substance abuse by PHC providers is key to its successful management. Administering self-reporting and substance abuse-screening tools in the course of a healthcare visit represents a screening strategy of secondary prevention.^19^ It is also critical for the department of education to early identify of children at risk of substance abuse through screening strategies.
- Tertiary prevention involves interventions to prevent the progression of established substance abuse before it has negative health consequences. Early intervention, pharmacological treatment, rehabilitation and social reintegration of substance abusers are part of the tertiary prevention.^19^

**FIGURE 2:**
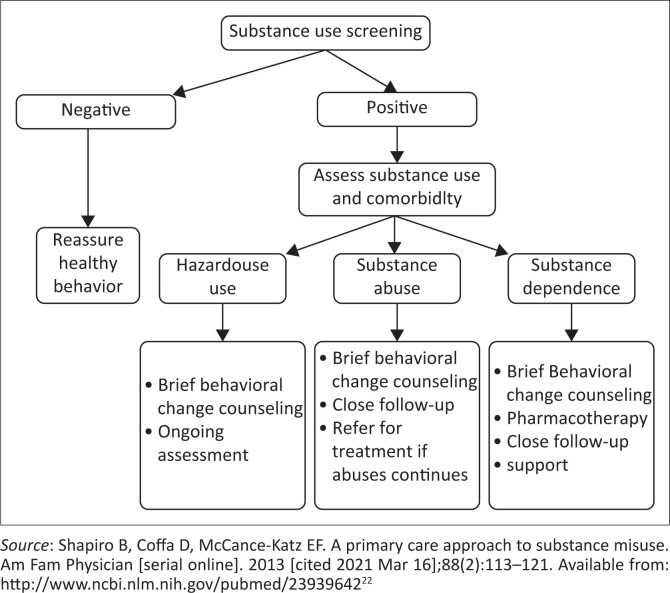
The management strategy of substance abuse.

The role of PHC providers in the prevention of substance abuse has been demonstrated by many research studies. Figure 2 illustrates some of the prevention strategies of substance abuse.

## Management

Substance abuse is an outcome of a complex interaction of hosts (substance abusers), agents (substances) and the environment (sociocultural context).^23^ The PHC provider’s approach to prevention and management of substance abuse should target susceptible hosts, substances and the creation of a supportive environment.^22^ Primary care is also well positioned to provide consultation on other psychosocial and behavioural problems that arise during the management of substance abuse.

A brief behavioural change counselling is readily available in primary care for substance abuse management. Evidence from research supports the premise that early intervention through a brief counselling is effective in achieving a long-term reduction in the use of alcohol, tobacco and other substances.^24^ The availability of pharmacotherapy intervention at primary care level can further enhance the management of substance abuse. Most substance abuse management occurs in the form of outpatient consultation in a primary care clinic. Primary healthcare settings providing such services in the context of healthcare can reduce stigma and increase patient access to appropriate substance abuse treatment.^25^ Close coordination between specialist consultation and PHC providers can achieve the desired outcomes of substance abuse management.

Patients with dual diagnoses and substance dependency need an appropriate referral for specialist care involvement. Dual diagnosis of psychiatric comorbidity is a most common diagnosis amongst substance abusers and the outcome depends on the primary diagnosis.^8^ Primary care is well equipped to deal with integrated management of the medical problems as well as psychiatric conditions amongst substance abusers.

## Conclusion

Primary care is the right place for routine screening and management of substance abuse. There are many good reasons to undertake substance abuse management within the domain of PHC. Substance abuse management within the context of primary care could increase access to and retention in care and improve health outcomes. The integrated care of substance abuse management can reduce the overall costs of healthcare. Social problems related to substance abuse, particularly for patients with chronic medical and mental health disorders, are well addressed in the PHC context. Specialist guidance in the coordinated care of substance abuse services in primary care settings can improve the continuum of healthcare. Similar to other chronic illnesses, substance abuse can be efficiently managed by PHC providers in a primary care structure by a brief behavioural counselling and medication. Indeed, the strength of primary care is in the use of PHC providers’ skills to address the broad range of healthcare issues related to substance abuse.
